# Supplementary material for: PSEN2 Thr421Met Mutation in a Patient with Early Onset Alzheimer’s Disease
Source: Int J Mol Sci. 2022 Nov 1;23(21):13331. doi: 10.3390/ijms232113331 (PMC9656741; doi:10.3390/ijms232113331)
Supplement: Supplementary file 1 [file ijms-23-13331-s001.zip › ijms-1932674-supplementary.pdf]

Supplement file:

Supplementary Table S1. Gene panel used in the analysis.

**Genes, used in the gene panel**
A2M, ABCA7, ABI3, ACTB, ACE, ADAM10, AIF1, AKT1, ALB, ALS2, ANG, APBB1, APCS, APH1A, APLP2, APOC2, APOC4,APOE, APP, ATP13A2, ATXN1, ATXN2, BACE1, BIN1, BST1. C9ORF72, CASP3, CASS4, CD2AP, CCNF, CD33, CDH12, CDH18, CDK5, CHAT, CHMP2B, CLU, CR1, CREB1, CSF1R , CST3, CTSB, CYP7B1, CTNNA3, CTSA, CTSD, DAO, DBN1, DCTN1, DLG4, DNMBP, DSG2, EPHA1, EWSR1, FBXO7, FERMT2, FIG4, FGF20, FUS, GAB2, GAK, GBA,GAPDH, GFAP, GIG25, GIGYF2, GPNMB, GRN, GPX4, GRIN2B, GRIN3B, GSK3A, GSK3B, HIP1R, HNRNPA1, HNRNPA2B1, HSPG2, HTRA1, HTT, IAPP, IDE, IL6, INS, LAMP3, LPR6, LRRK2, MAOB, MAPT, MARK4, MS4A4A, MEF2C, MS4A6E, MTND1, MTHFD1L, MTND2, NCSTN, NEFL, NEK1, NGF, NME8, NOTCH3, OPTN, PARK2, PARK7, PFN1, PHF1, PICALM, PILRA, PILRB, PINK1, PLA2G6 PLD3, PON1, PPP5C, PPT1, PRNP, PSEN1, PSEN2, PSENEN, PTK2B, PVRL2, RALGOS2, RBFOX3, RELB, RIN3, S100A9, SETX , SIRT1, SIGMAR1, SLC24A4, SNCA, SOD1, SORL1, SPAST, SPG11 SQSTM1, STBD1, STK39, STX1B, SYT11, SYNJ1, SYP, TAF15, TARDBP, TBK1, TM2D3, TMEM106B.TNF, TOMM40, TREM2, TREML2, TTR, UBQLN2, UNC5C, VAPB, VCP,VPS35, YWHAQ, ZCWPW1

Supplementary Table S2. Mutations, found in the patient by neuro-degenerative genetic analysis. PSEN2 Thr421Met was heighted with orange. Genes and variants, which were found to directly interact with PSEN2 Thr421Met through STRING were highlighted with yellow.

Gene	Mutation	RsID	1000g EAS	1000g ALL	gnomad_ exome_AF	gnomad_ex-ome_AF_eas	gnomad_ genome_All	gnomad_ genome_EAS	SIFT	Poly-Phen2
AARS2	p.I339V	rs324136	0.998	0.888978	0.9519	0.9985	0.9058	0.9974	0.378,T	0.0,B
ABCA13	p.P506L	rs1880738	0.6181	0.40655	0.449	0.5756	0.3982	0.6037	0.436,T	0.0,B
	p.R555H	rs2361519	0.1508	0.198083	0.1458	0.1688	0.1758	0.1603	1.0,T	0.002,B
	p.E741A	rs117022697	0.0655	0.013379	0.0051	0.0681	0.0037	0.0731	0.001,D	0.78,P
	p.I767S	rs17712293	0.2153	0.194489	0.1741	0.2245	0.1634	0.2063	0.007,D	0.294,B
	p.A2178E	rs1880736	0.9881	0.812899	0.7242	0.9893	0.7398	0.9929	1.0,T	0.0,B
	p.R2674W	rs2222648	0.9921	0.852236	0.8042	0.9921	0.7973	0.9942	1.0,T	0.0,B
	p.S3236G	rs184047949	0.006	0.001398	0.0012	0.0152	0.0007	0.0141	0.023,D	0.01,B
	p.N4277D	rs4917152	0.2163	0.230831	0.1731	0.2404	0.1871	0.221	1.0,T	0.0,B
	p.Q4302R	rs4917153	0.2163	0.230631	0.1731	0.2404	0.1874	0.2217	0.64,T	0.0,B
	p.H4343Q	rs113573154	0.006	0.001198	0.0007	0.0085	0.0005	0.0103	0.094,T	0.207,B
ABCA7	p.T319A	rs3752232	0.0923	0.112819	0.0548	0.0862	0.098	0.0954	0.882,T	0.0,B
	p.H395R	rs3764647	0.0923	0.108826	0.0534	0.0855	0.0942	0.0963	1.0,T	0.0,B
	p.R463H	rs3752233	0.1915	0.060703	0.0471	0.1784	0.0419	0.2077	0.254,T	0.989,D
	p.N718T	rs3752239	0.1855	0.059105	0.0473	0.1708	0.0419	0.2068	0.239,T	0.475,P
	p.R1349Q	rs3745842	0.3482	0.390575	0.4274	0.335	0.4203	0.3216	0.546,T	0.008,B
	p.G1527A	rs3752246	0.6478	0.825479	0.8396	0.646	0.8646	0.6723	0.877,T	0.0,B
	p.Q1686R	rs4147918	0.1736	0.05651	0.0478	0.1659	0.0418	0.195	0.234,T	0.0,B
ALS2	p.V368M	rs3219156	1	0.896565	0.9133	0.9998	0.8694	0.9994	0.191,T	0.006,B
ARAP2	p.R1523Q	rs4833069	1	0.995208	0.9922	1	0.9939	1	1.0,T	0.0,B
ATP13A2	p.A1072T	rs3170740	0.246	0.33127	0.4703	0.2946	0.4139	0.2865	0.988,T	0.001,B
ATXN1	p.Q214H	rs200111316	NA	NA	0.0034	0.0399	0.0058	0.0647	0.581,T	0.0,B
	p.H211Q	rs59310777	0.3879	0.266374	NA	NA	0.1189	0.6034	0.297,T	0.0,B
	p.H209Q	rs11969612	NA	NA	NA	NA	NA	NA	0.065,T	0.462,P
	p.H226de-linsQH	rs765686161	NA	NA	NA	NA	0.3449	0.1023	NA	NA
	p.Q216de-linsHQ	rs766029394	NA	NA	0.0039	0.0496	0.0056	0.0984	NA	NA

ATXN2	p.S248N	rs7969300	0.5228	0.179513	0.0781	0.563	0.068	0.5712	0.136,T	0.0,B
	p.188_189del	rs10560189	1	0.951078	NA	NA	0.8384	0.9303	NA	NA
BST1	p.R125H	rs2302465	0.0486	0.058506	0.0994	0.0351	0.0891	0.0256	0.124,T	1.0,D
CASS4	p.P660S	rs35031530	0.2381	0.146765	0.05	0.2522	0.091	0.2439	0.286,T	0.001,B
CD33	p.A14V	rs12459419	0.1855	0.210663	0.3074	0.184	0.2548	0.1787	0.029,D	0.766,P
CDH12	p.V68M	rs4371716	0.1438	0.36222	0.29	0.1443	0.3598	0.1277	0.454,T	0.0,B
CR1	p.H1658R	rs2274567	0.3274	0.294329	0.2504	0.2917	0.2048	0.2903	0.897,T	0.147,B
	p.T1858M	rs3737002	0.3264	0.248802	0.2825	0.3296	0.229	0.3295	0.019,D	0.987,D
	p.T2060S	rs4844609	1	0.995008	0.9855	1	0.9853	1	0.804,T	0.0,B
	p.I2065V	rs6691117	0.3433	0.493411	0.3214	0.3065	0.3957	0.314	1.0,T	0.0,B
	p.P2277R	rs3811381	0.3224	0.26278	0.2416	0.2886	0.1795	0.2851	0.446,T	0.005,B
	p.T2419A	rs2296160	0.6885	0.828075	0.8195	0.6536	0.8007	0.6576	0.987,T	0.0,B
CTNNA3	p.S596N	rs4548513	0.4187	0.485024	0.4043	0.4326	0.4818	0.4567	1.0,T	0.0,B
EPHA1	p.M900V	rs6967117	1	0.960264	0.9405	0.9984	0.9096	0.9994	1.0,T	0.0,B
	p.V160A	rs4725617	0.9921	0.94349	0.9329	0.9922	0.8915	0.9923	0.246,T	0.0,B
FAM105A	p.R349C	rs199613650	0.0089	0.001797	0.0008	0.0111	0.0007	0.0135	0.001,D	0.999,D
FBXO7	p.G6E	rs9621461	0.0615	0.065096	0.0896	0.0525	0.0821	0.0418	0.7,T	0.001,B
	p.M115I	rs11107	0.6915	0.48742	0.4526	0.6868	0.4099	0.6604	0.223,T	0.0,B
FIG4	p.M364L	rs2295837	0.1984	0.10004	0.0791	0.2068	0.0491	0.2358	1.0,T	0.0,B
FNDC1	p.T438A	rs509648	0.7232	0.508586	0.3252	0.7489	0.3866	0.7468	1.0,T	0.0,B
	p.E463Q	rs420137	0.621	0.784545	0.8661	0.6394	0.8419	0.6245	1.0,T	0.0,B
	p.Q1003E	rs370434	0.626	0.785743	0.8673	0.6433	0.8442	0.6265	1.0,T	0.0,B
	p.D1180E	rs420054	0.626	0.782348	0.8492	0.6242	0.8431	0.6289	1.0,T	0.0,B
	p.L1261P	rs3003174	0.626	0.786142	0.8684	0.6425	0.8443	0.6297	0.345,T	0.0,B
	p.Q1280R	rs2501176	0.626	0.786142	0.8689	0.645	0.8436	0.6247	1.0,T	0.0,B
	p.T1504K	rs386360	0.625	0.784944	0.8715	0.6464	0.8435	0.6276	0.795,T	0.0,B
	p.T1574A	rs7763726	0.3552	0.145567	0.0651	0.3175	0.0844	0.3451	0.042,D	0.15,B
	p.1471_1477 del	rs141435210	0.4058	0.496406	0.8698	0.656	0.8401	0.6223	NA	NA
GAK	p.K1265R	rs2306242	0.0863	0.035344	0.0431	0.0994	0.0361	0.0979	0.099,T	0.005,B
GAL3ST1	p.V29M	rs2267161	0.3502	0.309904	0.3028	0.3251	0.3216	0.3318	0.051,T	0.137,B
GBP4	p.E551G	rs561042	0.3978	0.357428	0.442	0.4022	0.4276	0.4198	0.036,D	0.095,B
	p.E551K	rs561037	0.3978	0.358826	0.4416	0.4017	0.4275	0.4191	0.505,T	0.001,B
	p.L549M	rs608339	0.3988	0.359425	0.4379	0.3986	0.428	0.4209	1.0,T	0.011,B
	p.E546K	rs1142890	0.3988	0.359026	0.4283	0.3891	0.427	0.4175	0.929,T	0.001,B
	p.M545I	rs1142889	0.3988	0.359026	0.427	0.3879	0.4269	0.418	0.072,T	0.004,B
	p.M545L	rs1142888	0.3988	0.359026	0.4259	0.3865	0.4267	0.4174	1.0,T	0.0,B
	p.M542I	rs1142886	0.3998	0.359625	0.4261	0.3851	0.4272	0.4174	1.0,T	0.0,B
	p.Y541N	rs655260	0.3998	0.359625	0.4306	0.3899	0.4276	0.4186	1.0,T	0.0,B
GIGYF2	p.P460T	rs2289912	0.2302	0.083067	0.0547	0.2251	0.0351	0.2143	0.133,T	0.519,P
	p.P1210fs	rs371622656	NA	NA	0.0464	0.1589	0.0553	0.261	NA	NA
	p.P1210fs	rs775324034	NA	NA	0.0339	0.1291	0.035	0.2137	NA	NA
HIP1R	p.V782M	rs2271051	0.2262	0.158546	0.0953	0.2355	0.1202	0.2526	0.53,T	0.0,B
HTRA1	p.A20V	rs369149111	0.1935	0.050719	0.0212	0.1542	0.0126	0.1388	0.633,T	0.001,B
LAMP3	p.I318V	rs482912	0.4712	0.496406	0.6493	0.5077	0.604	0.5322	1.0,T	0.0,B
LPA	p.L1372V	rs7765781	0.4107	0.414936	0.3549	0.3955	0.411	0.3811	0.967,T	0.073,B
	p.L1358V	rs7765803	0.4107	0.409145	0.3531	0.3961	0.4029	0.3821	1.0,T	0.15,B
LRP6	p.V1062I	rs2302685	0.9196	0.885583	0.8509	0.9331	0.8354	0.9377	1.0,T	0.0,B

LRRK2	p.R50H	rs2256408	1	0.969249	0.9928	1	0.9709	1	1.0,T	0.0,B
	p.S1647T	rs11564148	0.3373	0.285942	0.2976	0.3391	0.2884	0.351	0.953,T	0.0,B
	p.M2397T	rs3761863	0.4603	0.551717	0.6171	0.4677	0.6347	0.4779	0.466,T	0.0,B
MAPT	p.Y441H	rs2258689	0.628	0.312899	0.2818	0.632	0.2316	0.6354	0.978,T	0.001,B
MLXIP	p.E396G	rs7978353	0.3581	0.455871	0.4207	0.3851	0.4538	0.3854	0.506,T	0.0,B
NME8	p.C208R	rs10250905	0.5466	0.743411	0.7317	0.5622	0.7708	0.5674	0.046,D	0.001,B
NOTCH3	p.A2223V	rs1044009	0.5724	0.629393	0.7141	0.5846	0.7062	0.5794	0.175,T	0.001,B
	p.L1518M	rs141320511	0.0099	0.005791	0.0047	0.0069	0.002	0.0051	0.006,D	0.999,D
PDIA4	p.K79E	rs553265406	.	0.0002	1.59E-05	0.0002	NA	NA	0.554,T	0.594,P
PDLIM5	p.A374T	rs966845	1	0.993411	0.9938	1	0.9958	1	1.0,T	0.0,B
	p.T410A	rs7690296	0.3998	0.38758	0.4395	0.3979	0.37	0.3827	1.0,T	0.0,B
	p.S521N	rs13107595	1	0.979633	0.9897	1	0.9864	1	0.74,T	0.0,B
PSEN2	p.T421M	rs756609078	NA	NA	2.79E-05	0.0001	NA	NA	0.015,D	1.0,D
PTPRD	p.Q447E	rs10977171	0.121	0.050919	0.0455	0.1316	0.0332	0.1329	0.416,T	0.778,P
RIN3	p.T425M	rs3742717	0.4415	0.292532	0.2412	0.4665	0.1908	0.4682	0.08,T	0.293,B
	p.967_967del	rs570458246	NA	NA	0.6507	0.7902	0.6919	0.7941	NA	NA
SACS	p.V3369A	rs17078605	0.3145	0.258986	0.2866	0.3493	0.2138	0.3798	0.014,D	0.745,P
SETX	p.S2641G	rs3739927	0.3591	0.163538	0.0854	0.3858	0.0893	0.385	0.652,T	0.0,B
	p.I2616V	rs1056899	0.6835	0.538738	0.3826	0.7274	0.4449	0.7217	1.0,T	0.0,B
	p.T1855A	rs2296871	0.6379	0.443091	0.266	0.6949	0.3203	0.6849	0.83,T	0.0,B
	p.I1386V	rs543573	0.3621	0.55611	0.7345	0.3052	0.6805	0.3164	0.872,T	0.0,B
	p.G1252R	rs1183768	0.3621	0.55611	0.7344	0.305	0.6806	0.3183	0.133,T	0.796,P
	p.D1192E	rs1185193	0.3681	0.640575	0.7679	0.3127	0.7669	0.3252	0.377,T	0.004,B
	p.A660G	rs882709	0.4117	0.213658	0.1175	0.4479	0.1284	0.4394	0.008,D	0.728,P
SIGMAR1	p.Q2P	rs1800866	0.3204	0.217252	0.2013	0.3152	0.1681	0.3	0.343,T	0.0,B
SLC24A4	p.K533Q	rs45587635	0.1796	0.067292	0.0602	0.2281	0.0531	0.2194	0.184,T	0.059,B
SLC6A5	p.G102S	rs1443547	0.3651	0.405751	0.3697	0.3927	0.3676	0.3981	0.723,T	0.0,B
	p.F124S	rs1443548	0.75	0.793131	0.7714	0.7585	0.7902	0.7452	0.44,T	0.0,B
	p.A162G	rs1443549	1	0.99361	0.9986	1	0.9952	1	1.0,T	0.0,B
SMC5	p.V306I	rs1180116	0.8532	0.855032	0.8834	0.8552	0.8687	0.8456	0.655,T	0.0,B
SORL1	p.Q1074E	rs1699107	1	0.984824	0.996	1	0.9851	1	0.168,T	0.0,B
	p.V1967I	rs1792120	1	0.979433	0.996	1	0.9853	1	1.0,T	0.0,B
SPG11	p.F463S	rs3759871	0.4653	0.47484	0.4677	0.4558	0.483	0.4466	0.341,T	0.021,B
SYT11	p.Q48H	rs822522	1	0.954673	0.9907	0.9999	0.963	1	0.866,T	0.0,B
TET1	p.D162G	rs10823229	0.3919	0.258586	0.331	0.3834	0.2743	0.381	0.019,D	0.295,B
	p.S193T	rs12773594	0.1726	0.171725	0.1814	0.1714	0.169	0.1607	0.055,T	0.808,P
	p.A256V	rs12221107	0.1498	0.109425	0.1151	0.1509	0.0884	0.1408	0.094,T	0.022,B
	p.N1018S	rs16925541	0.1052	0.083267	0.1073	0.1163	0.0862	0.1173	0.09,T	0.024,B
	p.I1123M	rs3998860	0.8502	0.693291	0.7773	0.8403	0.7076	0.8383	0.157,T	0.07,B
TM2D3	p.L6R	rs2939587	1	0.993411	0.9795	1	0.9757	0.9994	0.311,T	0.0,B
TMEM106B	p.T185S	rs3173615	0.6508	0.595048	0.4906	0.658	0.4871	0.6596	0.214,T	0.043,B
VEPH1	p.S522P	rs11918974	0.3512	0.250599	0.2772	0.39	0.2191	0.3904	1.0,T	0.0,B
	p.V263G	rs1378796	0.2292	0.192692	0.1417	0.2421	0.1397	0.2206	0.463,T	0.0,B
	p.H34R	rs584115	0.7202	0.427915	NA	NA	0.3975	0.7526	1.0,T	0.0,B
	p.L35F	rs670526	0.9454	0.871006	NA	NA	0.8578	0.9544	1.0,T	0.0,B

Supplementary Table S3a. Genes which may be associated with PSEN2 by STRING networking

Gene	Disease	Function	Possible common pathway with PSEN2	Reference
ABCA7	AD	Lipid homeostasis, transport	Impacts amyloid trafficking, clearance, lipid homeostasis	PMID: 30903345
SORL1	AD	Endocytosis, sorting	Sorting receptor, involved in APP trafficking. May protect against amyloid processing. Impacts lipid homeostasis	PMID: 35457051
CASS4	AD	Tyrosine kinase, adhesive molecule	Amyloid metabolism, possible cytoskeletal function	PMID: 24951455
CD33	AD	Negative regulation of cytokine production, monocyte activation	Cell-cell communication, immune mechanism, amyloid phagocytosis	PMID: 21460840, PMID: 24951455
EPHA1	AD	protein-tyrosine kinase, involved in neuronal development	axon guidance, synaptic development and plasticity	PMID: 21460840, PMID: 24951455
SLC24A4	AD	sodium/calcium exchanger	Calcium transport	PMID: 34364289
NOTCH3	Vascular diseases	Intracellular signaling, neural development	Cleavage of Notch proteins by gamma secretase	PMID: 31502763
LRRK2	PD	Serine/threonine-protein kinase	Possible interaction through Wnt signaling	PMID: 31801553
MAPT	FTD	Microtubule assembly, stability	Tau modification, cytoskeleton organization	PMID: 19768372

Supplementary Table S3b. Genes which may be associated with PSEN2 by Cytoscape ClueGo networking. Genes, which may be associated directly with PSEN2 were highlighted with bold. Indirect association with PSEN2 through SORL1 and ABCA7 were also introduced

Gene	Disease	Function	Possible common pathway with PSEN2/impact in neurodegeneration	Reference
ABCA7	**AD**	**Lipid homeostasis, transport**	**Impacts amyloid trafficking, clearance, lipid homeostasis**	**PMID: 30903345**
SORL1	**AD**	**Endocytosis, sorting**	**Sorting receptor, involved in APP trafficking. May protect against amyloid processing. Impacts lipid homeostasis**	**PMID: 35457051**
HIP1R	**PD**	**phosphatidylinositol phosphate binding activity, signal transduction**	**May control the transport of growth factor receptors, impact neuronal development**	**PMID: 30574069**
LRP6	**AD**	**involved in endocytosis of lipoproteins and their ligands.**	**LRP6 is a coreceptor for Wnt signaling**	**PMID: 25242217**
SLC24A4	AD	sodium/calcium exchanger	Calcium transport	PMID: 34364289
LRRK2	PD	Serine/threonine-protein kinase	Possible interaction through Wnt signaling	PMID: 31801553
MAPT	FTD	Microtubule assembly, stability	Tau modification, cytoskeleton organization	PMID: 19768372

ATXN2	Ataxia, ALS	Involved in endocytosis modulates mTOR signals	Slows down Purkinje cell firing<br>Impact TDP43 misvocalization<br>Endocytosis related pathways	PMID: 29427103<br>PMID: 20740007
GAK	PD	Regulates the cyclin related pathways	May impact protein clearance, autophagy, lysosome functions	PMID: 31783880
SETX	ALS, ataxia	Transcription regulation, splicing	Impact the regulation of autophagy	PMID: 31783880
VPS11	ALS	Impact endosome-lysosome functions	Impact the regulation of autophagy	PMID: 34920755
SYT11	PD	Impact synaptic transmission, membrane transmission	Cytokine secretion inhibition, microglia phagocytosis<br>May regulate amyloid peptide generation	PMID: 28686317<br>PMID: 26202512
TMEM106B	FTD	ATPase, dendrite morphogenesis, lysosome localization	Regulator of lysosome functions, neuronal development	PMID: 27543298
SACS	ataxia	chaperon	Protein folding, microtubule organization, mitochondria, autophagy	PMID: 35008978
